# Quantitative analysis of recombination between YFP and CFP genes of FRET biosensors introduced by lentiviral or retroviral gene transfer

**DOI:** 10.1038/srep13283

**Published:** 2015-08-20

**Authors:** Akira T. Komatsubara, Michiyuki Matsuda, Kazuhiro Aoki

**Affiliations:** 1 Laboratory of Bioimaging and Cell Signaling, Graduate School of Biostudies, Kyoto University, Sakyo-ku, Kyoto 606-8501, Japan; 2 Department of Pathology and Biology of Diseases, Graduate School of Medicine, Kyoto University, Sakyo-ku, Kyoto 606-8501, Japan; 3 Imaging Platform for Spatio-Temporal Information, Graduate School of Medicine, Kyoto University, Sakyo-ku, Kyoto 606-8501, Japan

## Abstract

Biosensors based on the principle of Förster (or fluorescence) resonance energy transfer (FRET) have been developed to visualize spatio-temporal dynamics of signalling molecules in living cells. Many of them adopt a backbone of intramolecular FRET biosensor with a cyan fluorescent protein (CFP) and yellow fluorescent protein (YFP) as donor and acceptor, respectively. However, there remains the difficulty of establishing cells stably expressing FRET biosensors with a YFP and CFP pair by lentiviral or retroviral gene transfer, due to the high incidence of recombination between *YFP* and *CFP* genes. To address this, we examined the effects of codon-diversification of YFP on the recombination of FRET biosensors introduced by lentivirus or retrovirus. The *YFP* gene that was fully codon-optimized to *E.coli* evaded the recombination in lentiviral or retroviral gene transfer, but the partially codon-diversified YFP did not. Further, the length of spacer between *YFP* and *CFP* genes clearly affected recombination efficiency, suggesting that the intramolecular template switching occurred in the reverse-transcription process. The simple mathematical model reproduced the experimental data sufficiently, yielding a recombination rate of 0.002–0.005 per base. Together, these results show that the codon-diversified YFP is a useful tool for expressing FRET biosensors by lentiviral or retroviral gene transfer.

Biosensors based on the principle of Förster (or fluorescence) resonance energy transfer (FRET) have shed new light on the spatiotemporal dynamics of signalling molecules in a living cell. The FRET biosensors are largely grouped into intermolecular and intramolecular FRET biosensors. A number of intramolecular FRET biosensors, which comprise both the donor and the acceptor fluorophores within a single protein, have been developed to visualize signalling molecules such as Ca^2+^^1^, phospholipids^2^,^3^, small GTPases^4^, protein kinases^5^ and so on^6^,^7^. It is widely accepted that the intramolecular FRET biosensors enjoy higher sensitivity and easier loading to cells and mice as compared with intermolecular FRET biosensors, which consist of a pair of donor and acceptor fluorophores^8^,^9^.

A critical drawback of the intramolecular FRET biosensors is that conventional gene-delivery techniques including the transfection of linearized DNAs and viral vectors of Retroviridae often fail to generate stable cell lines expressing FRET biosensors^10^. In many cases, the generated cell lines express only the donor or acceptor fluorescent protein. This phenomenon may be due to recombination between the donor and acceptor fluorescent proteins. We recently found that *piggyBac*^11^ or *Tol2*^12^ transposon-mediated gene transfer can be used to establish cell lines and transgenic mice expressing FRET biosensors^13^,^14^. Live cell FRET imaging of up to several days has revealed the fluctuation of signalling molecules such as ERK and Rac1^10^,^13^,^15^. The transposon-mediated gene transfer technique, however, is applied only to cells into which plasmids are efficiently transfected by lipofection or electroporation. In contrast, retroviral vectors can infect a wide range of cells *in vitro* and *in vivo*.

It is known that recombination occurs between homologous RNA sequences in the process of retrovirus-mediated gene transfer^16^,^17^. Among several models of such recombination, the most widely accepted is the template switching model. In this model, RNA-dependent DNA polymerase, i.e., reverse transcriptase, jumps from one template RNA to the other of the two single-stranded genomic RNAs in each retrovirus. The high sequence similarity is considered to guide the template switching. Currently, most intramolecular FRET biosensors adopt a cyan fluorescent protein (CFP) and a yellow fluorescent protein (YFP) as the donor and acceptor fluorescent proteins, respectively. Because both CFP and YFP are derived from GFP, they share high nucleotide sequence homology, which presumably causes the recombination. Consistent with this idea, tandem *GFP* genes or *tandem-dimer Tomato* (*tdTomato*) genes have shown clear recombination during the process of lentiviral or retroviral gene transfer^18^,^19^. Further evidence is provided by the results that FRET biosensors carrying a YFP and a coral-derived teal fluorescent protein (TFP) are readily expressed by retrovirus-mediated gene transfer without any recombination^20^,^21^,^22^. The nucleotide sequence homology between codon-humanized *TFP* (*hTFP*) and codon-humanized *YFP* (*hYFP*) (61%) is markedly lower than the homology between codon-humanized *CFP* (*hCFP*) and *hYFP* (96%) (Supplementary Fig. S1). Although TFP has some advantages over CFP as a FRET donor^23^, the substitution of CFP to TFP decreases FRET gain more than in most FRET biosensors containing YFP as the acceptor, which could be due to the weak or absent dimerization of YFP and TFP^13^.

In consideration of these facts, a few research groups have successfully utilized a pair of codon-diversified *YFP* mutant and *CFP* to establish stable cell lines expressing FRET biosensors by retroviral transduction^22^,^24^. However, there have been no reports analyzing the effect of codon diversification on the efficiency of recombination in FRET biosensors transduced by retrovirus systematically. Therefore, we examined recombination in FRET biosensors with codon-diversified *YFP* mutants delivered by two retroviral vectors, a Murine leukemia virus (MuLV)-derived pCX4 retroviral vector^25^ and a human immunodeficiency virus (HIV)-derived pCSII lentiviral vector^26^. In addition, based on the experimental data, we evaluated the recombination rate in lentiviral or retroviral gene transfer by mathematical modelling and statistical analysis.

## Results

### Construction of codon-diversified YFP genes

**Figure 1:**
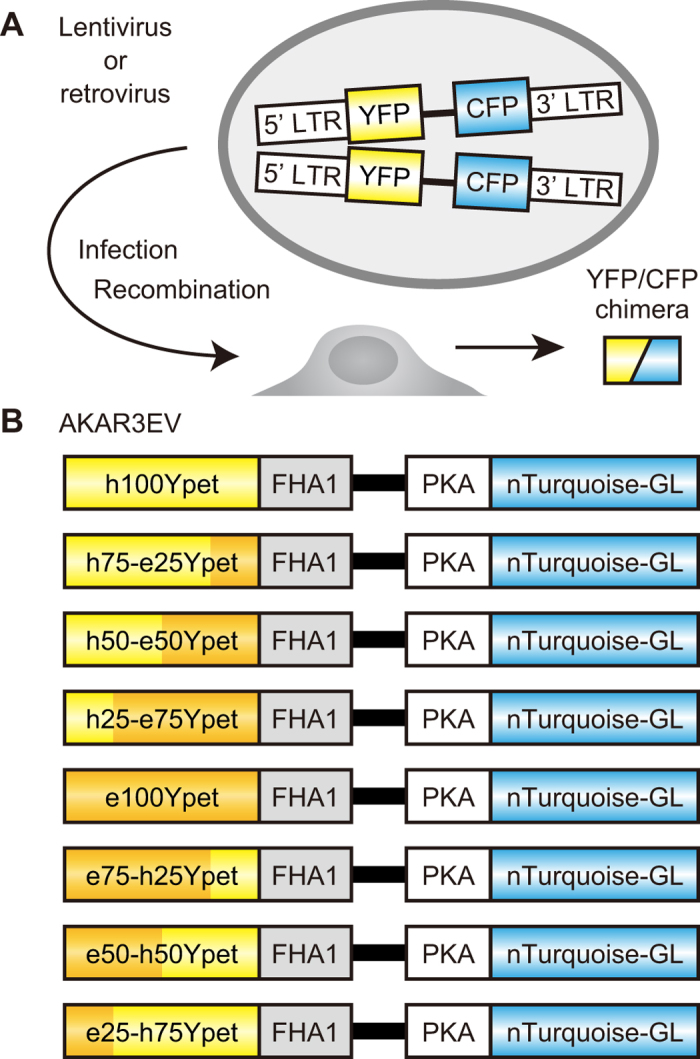
Recombination of FRET biosensors during lentiviral or retroviral infection. (**A**) Schematic representation of the recombination between *YFP* and *CFP* genes in FRET biosensors in the process of lentiviral or retroviral gene transfer. Two copackaged genomic RNAs encoding FRET biosensors are included in a virus particle. After infection, cells express only YFP or CFP. (**B**) FRET biosensors with different YFP variants. A PKA FRET biosensor, AKAR3EV, is composed of YPet (YFP), a FHA1 domain, linker, PKA substrate, nTurquoise-GL (CFP), and a nuclear export sequence (NES). In this study, YPet is replaced with *h100YPet*, *H75-e25YPet*, *h50-e50YPet*, *h25-e75YPet*, *e100YPet*, *e75-h25YPet*, *e50-h50YPet*, and *e25-h75YPet*.

We used a FRET biosensor for protein kinase A, AKAR3EV^13^, to examine the contribution of nucleotide sequence homology in recombination between CFP and YFP (Fig. 1A). AKAR3EV comprised a YFP-derived YPet^27^, and a CFP-derived nTurquoise-GL^28^ as the acceptor and donor, respectively. These fluorescent proteins sandwich the phosphate-binding domain of FHA1, EV linker, and a substrate peptide of PKA (Fig. 1B). The nuclear export signal (NES) was included at the C-terminus of the biosensor. Both the *YPet* and the *nTurquoise-GL* genes have been codon-optimized for humans. The homology between the humanized *YPet, called hYPet hereafter,* and *nTurquoise-GL* was 96%. As the codon-diversified *YFP*, we chose a *YPet* gene optimized for E. coli, called *eYPet* hereafter. The nucleotide sequence homology between *eYPet* and *nTurquoise-GL* was 68% (Supplementary Fig. S1). We constructed six *YPet* chimeras between *hYPet* and *eYPet: h75-e25YPet*, *h50-e50YPet*, *h25-e75YPet*, *e75-h25YPet*, *e50-h50YPet*, and *e25-h75YPet* (Fig. 1B, and see Methods). The order of *h* and *e* and their numbers indicated the order and the percentage ratio of *hYPet* to *eYPet*, respectively. For instance, *h75-e25YPet* was composed of the first 75% of the *hYPet* gene DNA sequence, followed by the last 25% of the *eYPet* gene DNA sequence. *h100YPet* and *e100YPet* are identical to the authentic *hYPet* and *eYPet*, respectively. These genes for FRET biosensors were inserted into either the MuLV-derived retroviral vector or HIV-derived lentiviral vector, which were transfected into 293T cells to generate retroviral or lentiviral vectors.

### A template-switching model for the gene recombination during lentivirus - or retrovirus-mediated gene transfer

**Figure 2:**
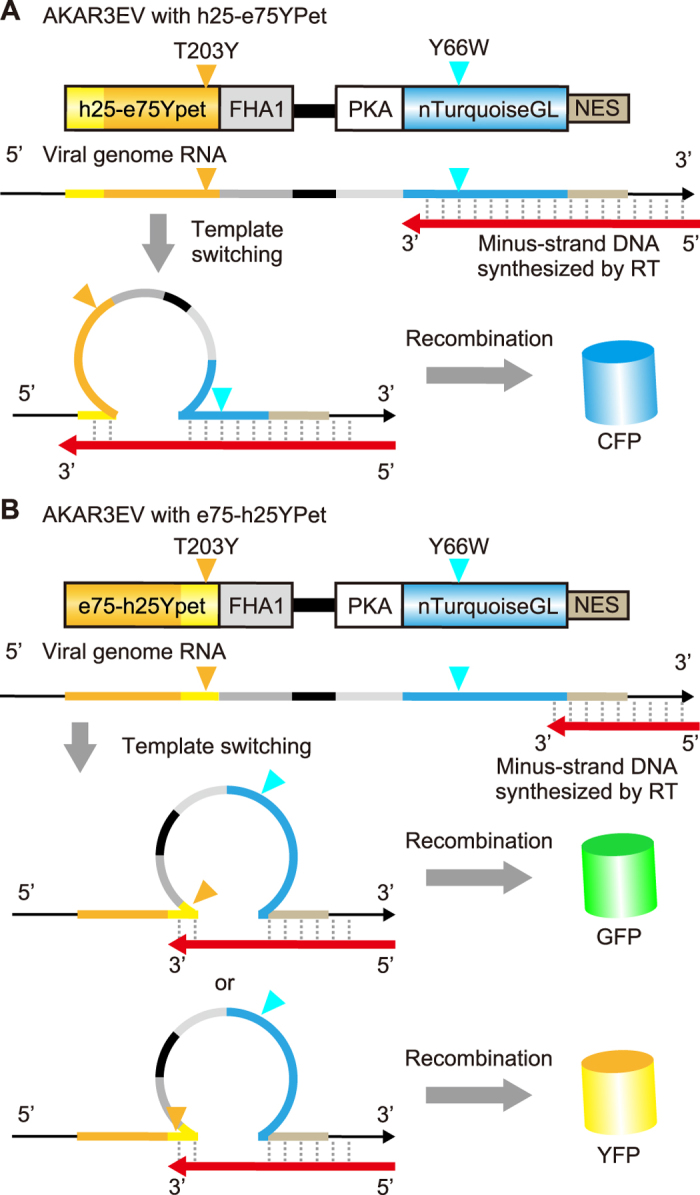
Working model for the recombination in FRET biosensors. (**A**) The recombination of FRET biosensors with *h25-e75YPet* generates CFP, which includes the critical amino acid substitution of Y66W from GFP. (**B**) The recombination of FRET biosensors with *e75-h25YPet* generates GFP or YFP, which includes the critical amino acid substitution of T203Y from GFP.

Recombination of retroviral genomes could be caused by template switching/jumping during minus-strand DNA synthesis, or reverse transcription, on the RNA genome and plus-strand DNA replication. Figure 2 shows the case of template switching/jumping during reverse transcription. This model provided a proper interpretation of the results with the series of lentiviral or retroviral vectors: Assuming that *e100YPet* is not recombined with *nTurquoise-GL* and that the hues of YFP and CFP are determined primarily by the T203Y substitution and the Y66W substitution, respectively^29^, we can expect the outcomes of recombination between the *YPet* chimeras and *nTurquoise-GL* illustrated in Fig. 2: First, *h25-e75YPet* is recombined with *nTurquoise-GL* in the first-quarter segment. In this case, the recombined fluorescent protein gene will carry the nucleotides for Y66W, but not for T203Y, and therefore encodes CFP (Fig. 2A). Second, *e75-h25YPet* is recombined with *nTurquoise-GL* in the fourth-quarter segment. In this case, the resulting fluorescent protein will miss the nucleotides for Y66W and may or may not contain the nucleotides for T203Y, and therefore encodes either *GFP* or *YFP* (Fig. 2B). The recombination between *YPet* and *nTurquoise-GL* generate 4 types of chimeric GFP (Supplementary Fig. S2A and S2B). It is expected that chimeric GFP with H148G mutation fluoresced less than the wild-type GFP and YFP, because of the higher *pK*_a_ value^30^. In fact, we confirmed that the chimeric GFP emitted less fluorescence than original YPet in our experimental condition (Supplementary Fig. S2B). Therefore, the decrease of fluorescence was caused by substation the *nTurquoise-GL* sequence for *YPet* sequence.

### Recombination between YFP and CFP genes expressed by the lentiviral vector

We infected cells of the human lung adenocarcinoma cell line A549 with HIV-derived viruses carrying one of the eight FRET biosensor genes at a multiplicity of infection (MOI) of approximately 0.7. Approximately one week after infection without drug selection, the cells were imaged with epi-fluorescence microscopy. The fluorescence intensities of YFP and CFP in each cell were quantified and are shown in scatter plots.

**Figure 3:**
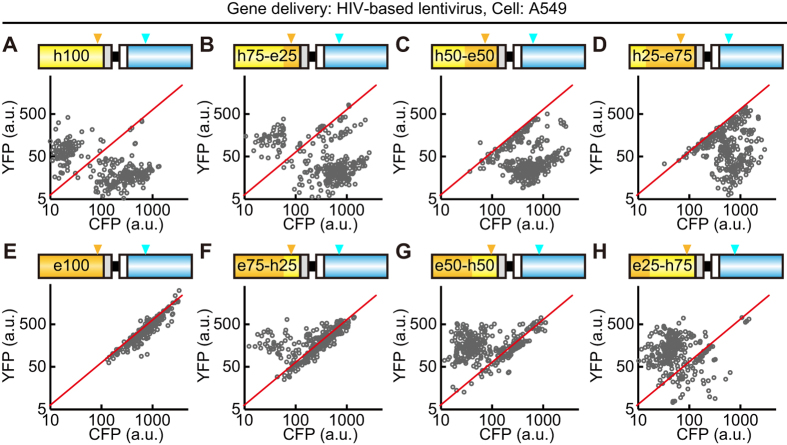
Recombination between the *YFP* and *CFP* genes by lentiviral gene transfer. (**A**–**H**) A549 cells were infected with lentivirus encoding 8 different FRET biosensors as shown in Fig. 1B. At least 4 days after infection, the cells were imaged with an epi-fluorescence microscope. The average fluorescence intensities of CFP and YFP are represented as a log-log plot. Each dot corresponds to an A549 cell. Three hundred cells were analyzed from two independent experiments. Red lines are the fitted line with the *e100YPet* data. Orange and cyan arrowheads indicate the T203Y and Y66W positions, respectively.

Most cells infected with the *h100YPet-*carrying lentivirus demonstrated clear evidence of recombination between the *YFP* and *CFP* genes, i.e., cells expressed either YFP or CFP, but not both (Fig. 3A). In cells infected with the *h75-e25YPet-*carrying virus, the fraction of cells emitting YFP fluorescence alone was smaller than that in *h100YPet-*carrying virus-infected cells, and cells emitting both CFP and YFP fluorescences were detectable (Fig. 3B). In cells infected with the *h50-e50YPet-*carrying virus or *h25-e75YPet-*carrying virus, all cells emitted either CFP fluorescence alone or both CFP and YFP fluorescences (Fig. 3C,D). As expected, the *e100YPet*-carrying lentivirus did not show any sign of recombination and emitted equal amounts of CFP and YFP fluorescence (Fig. 3E). In cells infected with the *e75-h25YPet-*carrying virus, a small fraction of cells emitted YFP fluorescence alone (Fig. 3F). The fraction of cells that emitted YFP fluorescence alone was increased in the e50*-h50YPet-*carrying virus and e25*-h75YPet-*carrying virus, concomitant with the decrease in cells emitting both CFP and YFP fluorescences (Fig. 3G,H). Of note, the fluorescence intensity of chimeric fluorescent proteins was less than the prototype as described; the average intensities of YFP channel was highest in the prototype e100YPet-carrying virus (Fig. 3E–H). Overall, very similar results were obtained when HeLa cells were infected at an MOI of less than 1.0 (Supplementary Fig. S3A–S3H). These results are in line with the model shown in Fig. 2, supporting the idea that the recombination is caused by template switching/jumping during reverse transcription.

### Recombination between YFP and CFP genes expressed by the retroviral vectors

**Figure 4:**
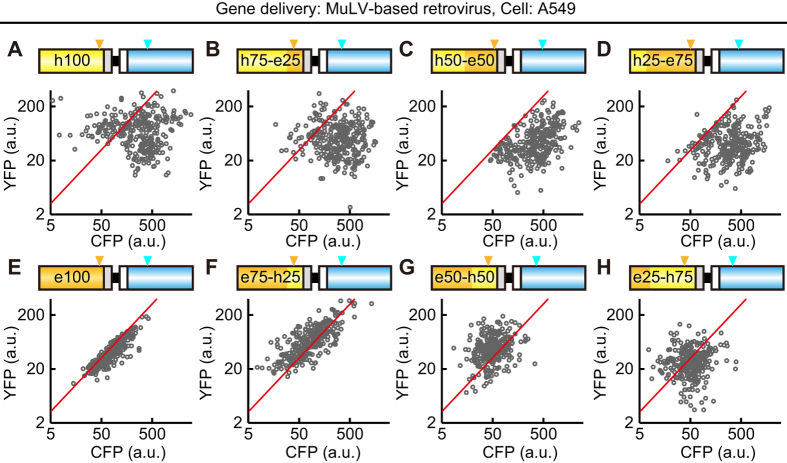
Recombination between the *YFP* and *CFP* genes by retroviral gene transfer. (**A**–**H**) A549 cells were infected with retrovirus encoding 8 different FRET biosensors as shown in Fig. 1B. At least 4 days after infection, the cells were imaged with an epi-fluorescence microscope. The average fluorescence intensities of CFP and YFP are represented as a log-log plot. Each dot corresponds to an A549 cell. Three hundred cells were analyzed from two independent experiments. Red lines are the fitted line with the *e100YPet* data. Orange and cyan arrowheads indicate the T203Y and Y66W positions, respectively.

We then used a MuLV-derived retroviral vector to express the FRET biosensors stably, and analyzed the results as described above (Fig. 4). The MOI was approximately 0.5. Cells infected with the *h100YPet-*carrying retrovirus did not show clear cell populations, although significant fractions of the cells emitted CFP fluorescence much more strongly than they did YFP fluorescence (CFP-dominant cells), or emitted YFP fluorescence much more strongly than they did CFP fluorescence (YFP-dominant cells) (Fig. 4A). In cells infected with the *h75-e25YPet-*carrying virus, the CFP-dominant cells were increased with a concomitant decrease in YFP-dominant cells (Fig. 4B). In cells infected with the *h50-e50YPet-*carrying virus or *h25-e75YPet-*carrying virus, the CFP-dominant cells were further increased with the disappearance of YFP-dominant cells (Fig. 4C,D). Again, the *e100YPet -*carrying retrovirus did not show any sign of recombination and emitted equal amounts of CFP and YFP fluorescence (Fig. 4E). The YFP-dominant cells appeared in cells infected with the *e75-h25YPet-*carrying virus (Fig. 4F) and were further increased in those infected with the e50*-h50YPet-*carrying virus (Fig. 4G). Cells infected with the e25*-h75YPet-*carrying virus did not exhibit any clear subpopulations (Fig. 4G,H).

When using HeLa cells as a host, we obtained similar but slightly different results (Supplementary Fig. S3I–S3P). For example, the *e100YPet-*carrying retrovirus also showed some sign of recombination; i.e., the appearance of CFP-dominant cells. These observations suggested that the recombination was affected to some extent by viral vectors, and/or host cells. Finally, we confirmed that the recombination was not observed in cells transfected with the expression plasmids used for the preparation of lentivirus and retrovirus (Supplementary Fig. S4).

### The effect of the spacer between *YFP* and *CFP* on the recombination

**Figure 5:**
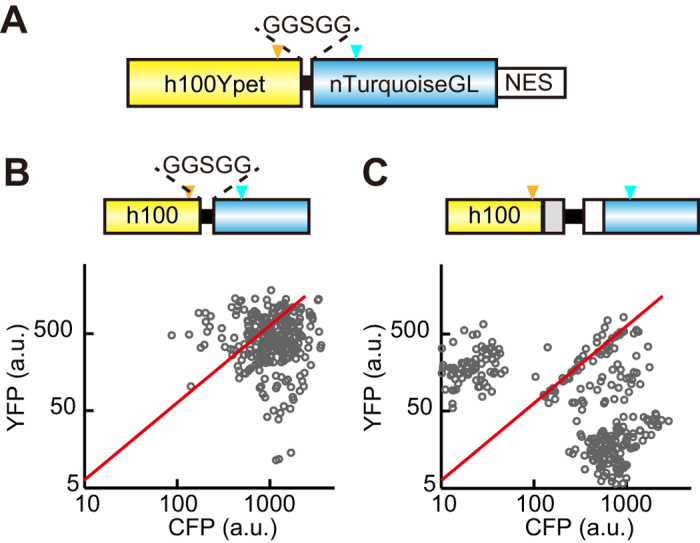
Effect of a short spacer between *YFP* and *CFP* on recombination. (**A**) Schematic representation of a FRET biosensor with YPet, GGSGG linker, nTurquoise-GL, and NES. (**B**,**C**) HeLa cells were infected with lentivirus encoding FRET biosensor with a short spacer, 15 bases (**B**) and full spacer, 812 bases (**C**). At least 4 days after infection, 300 cells were imaged with an epi-fluorescence microscope, and represented as in Fig. 3. Note that panel C is the same graph as in Supplementary Fig. 2A. Orange and cyan arrowheads indicate the T203Y and Y66W positions, respectively.

We next examined whether the length of the spacer between *YFP* and *CFP* affected the recombination. For this purpose, we developed an *h100YPet*-carrying lentiviral vector, in which *h100YPet* and *nTurquoise-GL* were linked with a GGSGG linker (15 bases) (Fig. 5A). Cells infected with the *h100YPet-GGSGG-nTurquoise-GL-carrying* lentivirus exhibited markedly less, but not negligible, recombination (Fig. 5B) than did those infected with the *h100YPet*–carrying lentivirus, in which the length between the two fluorescent genes was 812 bases (Fig. 5C). This result may suggest that the recombination took place in a single copy of the lentiviral gene, rather than between the 2 copies of the lentiviral gene in a single virus (see Discussion).

### The validation of the recombination

To validate the recombination between *YPet* and *nTurquoise-GL* genes, we sequenced the viral DNAs integrated into the genomes. We sorted A549 cells infected with *h100YPet*-carrying lentivirus depending on the fluorescence of YFP, followed by genomic DNA extraction, PCR amplification of the recombined fluorescent protein genes, and sequencing. As expected, recombination was found between *YPet* and *nTurquoise-GL* genes (Supplementary Fig. S5A).The frequency of recombination was apparently correlated with the length of homology regions in between the differences of *YPet* and *nTurquoise-GL* genes (Supplementary Fig. S5B and S5C). However, the recombination in 448–609 base region occurred more frequent considerably than in the other regions. These results suggested that recombination took place almost randomly in between the identical nucleotide sequences, and the secondary structure of RNA viral genomes affected the recombination frequency to some extent.

### Mathematical modelling of recombination

Finally, we attempted to quantify the recombination rate by mathematical model and statistical analysis. The following two assumptions were made in the computer simulation: First, based on the results in Supplementary Figure S5, we assumed that the *CFP* (*nTurquoise-GL)* gene is recombined randomly with the identical nucleotide of *hYFP*, but not *eYFP*. Second, one copy of the retroviral gene is integrated into the genome of the host cells under each condition. The mathematical model included only one parameter to be fitted—the recombination rate, *r* (/base), which represented the probability of recombination per base. The parameter *r* was obtained by the maximal likelihood estimation method (for more details, see Methods and Supplementary Fig. S6).

**Figure 6:**
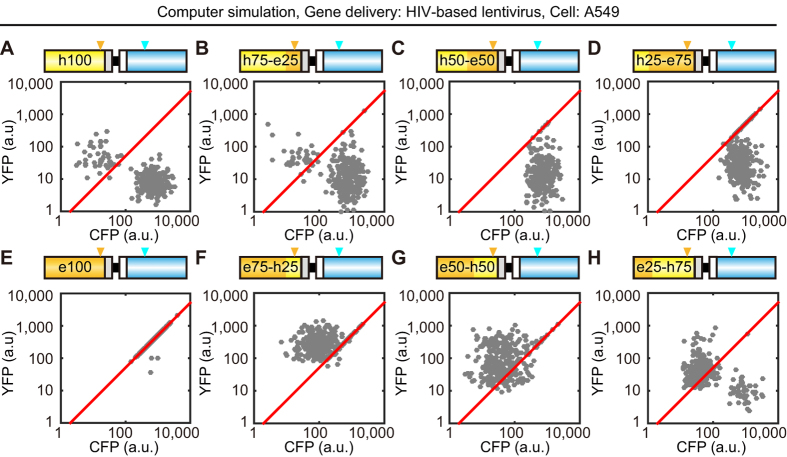
Computer simulation of recombination between the *YFP* and *CFP* genes. The recombination of *YFP* and *CFP* genes in A549 cells infected with the indicated lentivirus was simulated by computer with a recombination rate of 0.0043 (/base), which showed maximal likelihood estimation. To reproduce the experimental data, 9 parameters were extracted from the experimental data set in Fig. 3 (see Supplementary Fig. S6 and the Methods for details). Red lines were fitted with the *e100YPet* data. Orange and cyan arrowheads indicate the T203Y and Y66W positions, respectively.

The simple mathematical model qualitatively reproduced the four experimental data sets of A549 cells and HeLa cells infected with lentiviral or retroviral vectors (Fig. 6 and Supplementary Fig. S7). These data validated our hypothesis that the recombination was governed by a random process within the homology region. Interestingly, the recombination rate of lentivirus did not differ from that of retrovirus; both were 0.002 ~ 0.005 /base (Table 1 and Supplementary Fig. S8). Further, the lentivirus with shorter spacer between *YFP* and *CFP* genes indeed showed smaller recombination rate than that with longer spacer (Table 1).

## Discussion

Here, we demonstrated that a FRET biosensor comprised of *e100YPet* and *nTurquoise-GL* could be applied to lentivirus- or retrovirus-mediated gene transfer, and such transfer had the lowest possibility of recombination when performed in HeLa cells and A549 cells. Previous studies have proven the impact of codon usage on the gene expression^31^, and therefore we expected that the substitution of *h100YPet* partly or totally by *e100YPet* would decrease the expression of the FRET biosensor. Unexpectedly, we did not detect any difference in the fluorescence intensity between the FRET biosensors codon-optimized for humans and bacteria (Figs 3,4, and Supplementary Figs S3, S4). Because the *YFP* transcript is relatively short (714 bases), the effect of codon usage bias may not substantially affect the expression level of the FRET biosensor. Furthermore, considering the fact that the difference in cell lines did not change the results of recombination in the series of FRET biosensors, we could conclude that *e100YPet* enabled us to establish stable cell lines expressing highly sensitive FRET biosensors with the YPet and CFP pair^13^ by lentivirus- or retrovirus-mediated gene transfer.

The finding that the recombination rate was reduced when we used a short spacer between *h100YPet* and *nTurquoise-GL* (Fig. 5) may suggest that the recombination between the *YFP* and *CFP* genes occurs by template switching within a single RNA genome but not between the two RNA genomes in a single retroviral particle. Because the template switching within the RNA template is expected to suffer from steric hindrance, the shorter spacer between *h100YPet* and *nTurquoise-GL* may dampen the recombination efficiency. There is additional evidence that supports the template switching within a single RNA genome. If the template switching between the two RNA genomes occurred, then the *h25-e75YPet-*carrying virus should generate not only cells expressing CFP alone, but also cells expressing the biosensor with two copies of YFP and a single copy of CFP. However, we could not detect such cells by quantitative imaging (Figs 3D and 4D). Hu *et al.* reported that deletion of repeated regions occurs by an intramolecular template switch within the same RNA in the process of reverse transcription^32^. The rate of intramolecular recombination is much more efficient than that of intermolecular recombination, and the value of the intramolecular recombination rate has been roughly estimated as 30–41% per 110 bases, and thus 0.27–0.37% per base^32^,^33^. In agreement with these facts, these values were almost identical to our data (Table 1), strongly supporting the notion that the intramolecular recombination took place in FRET biosensors transduced by lentivirus or retrovirus. Even with the aforementioned evidence, we still could not completely neglect the template switching between the two RNA genomes. A potential merit of template switching between the two RNA genomes is to short-cut the reverse transcription in a viral particle. Because Hu *et al.* examined the recombination in a 110-bp direct repeat, there is little advantage to the minus-strand DNA synthesis. This idea might also explain why shortening of the spacer between *YPet* and *nTurquoise-GL* decreased the rate of recombination.

We observed the recombination even in the FRET biosensor with *h25-e75YPet* or *e75-h25YPet*, which shares high nucleotide sequence homology with *nTurquoise-GL* only in the quarter of the *YPet* genome (Figs 3,4 and Supplementary Figs S3, S4). This is also consistent with the model of template switching, because the reverse transcriptase jumps to the other RNA template through the binding of minus-strand DNA to the complementary RNA^18^. Although we do not know the length of homologous DNA sequence required, a length of at least 178 bases, which corresponds to 25% of the *YFP* gene, is sufficient for the recombination mediated by lentiviral or retroviral gene transfer. Salamongo *et al.* have reported that template jumping occurred between the short identical sequences (21 bases) at both the N-terminal and C-terminal sequences in *GFP* and *Tomato*^19^, suggesting that codon diversification, but not domain swapping, is necessary to avoid recombination.

In conclusion, here we have shown that, in addition to transposon-mediated gene transfer, lentivirus- or retrovirus-mediated gene delivery is also available for establishing a stable cell line expressing FRET biosensors with the YFP and CFP pair. This technique will provide new insight into not only the effect of molecular-targeted drugs on the signalling molecules^13^, but also intravital imaging in living animals.

## Methods

### FRET biosensor construction

The FRET biosensors developed in this study were originated from pAKAR3EV, a PKA biosensor^13^. To generate *e100YPet*, *h75-e25YPet*, *h50-e50YPet*, *h25-e75YPet*, *e75-h25YPet*, *e50-h50YPet*, and *e25-h75YPet*, the YPet-based Rac1 biosensor Raichu-Rac1, which was a kind gift from Dr. Wang of University of California at San Diego^34^ and contained *e100YPet*, was used as a PCR template. The *hYPet* gene in the original pAKAR3EV was replaced with these *YPet* variants. These FRET biosensor genes were inserted into pCX4bsr^25^ or pCSIIbsr, which was derived from pCSII-EF1 (a kind gift from Dr. Miyoshi, RIKEN) with IRES-*bsr* (blasticidin S-resistant gene).

### Cell culture

HeLa cells were purchased from the Human Science Research Resources Bank (Sennanshi, Japan). HEK-293T cells were obtained from Invitrogen as Lenti-X 293 cells (Carlsbad, CA). A549 cells were obtained from the American Tissue Culture Collection. HeLa, HEK-293T, and A549 cells were maintained in DMEM (Wako, Osaka, Japan) supplemented with 10% FBS. For imaging, these cells were plated on 35-mm glass base dishes (Asahi Techno Glass, Tokyo, Japan). One hour before observation, HeLa and A549 cells were maintained with phenol red-free Medium 199 (Invitrogen) containing 0.1% bovine serum albumin and 20 mM HEPES.

### Lentivirus or retrovirus-mediated gene transfer

For lentiviral production, HEK-293T cells were cotransfected with the pCSIIbsr vector, psPAX2 (Addgene plasmid 12260), and pCMV-VSV-G-RSV-Rev by using Polyethyleneimine “Max” MW 40,000 (Polyscience Inc., Warrington, PA). For retrovirus production, pCX4bsr, pGP, and pCMV-VSV-G-RSV-Rev were introduced into HEK-293T cells. Virus-containing media were collected at 48 hours after transfection, filtered, and used to infect target cells with 8 μg/mL polybrene. At least 4 days after infection, the infected cells without drug selection were imaged with a fluorescence microscope.

### Spectroscopy by fluorescence microscope and analysis

CFP and YFP images of HeLa cells and A549 cells were obtained by using an inverted microscope (IX81-ZDC; Olympus, Tokyo, Japan) equipped with a cooled CCD camera (Cool SNAP-K4; Roper Scientific), an illumination system (Spectra-X light engine; Lumencore, OR), an IX2-ZDC2 laser-based autofocusing system (Olympus), a MAC5000 controller for filter wheels and XY stage (Ludl Electronic Products, Hawthorne, NY), an incubator chamber system (Tokai Hit, Shizuoka, Japan) and a GM-4000 CO2 supplier (Tokai-Hit, Fujinomiya, Japan). The following filters were used for the dual emission imaging studies: FF01–438/24–25 (Semrock, Rochester, NY, USA), and FF01–475/28–25 (Semrock) excitation filter for CFP and YFP/GFP, a U-MREF glass reflector (Olympus) as a dichroic mirror, an FF01–483/32–25 emission filter (Semrock) for CFP and an FF01–542/27–25 emission filter (Semrock) for YFP. The microscope was controlled by MetaMorph software (Universal Imaging, West Chester, PA). The average fluorescence intensities of CFP and YFP in each cell were measured by manually delineating a region of interest at the cytoplasm with MetaMorph software.

### FACS and purification of genomic DNA

A549 cells infected with *h100YPet*-carrying lentivirus were sorted with a FACSAria™ III (BD Biosciences). YFP/CFP fluorescences were detected using a 445 nm laser, a 530/30 nm emission filter for YFP and a 480/20 nm emission filter for CFP. The genomic DNAs of these bulk cells were extracted by QuickExtract™ DNA Extraction Solution (Epicentre, Madison, WI, USA) in accord with manufacture’s protocol. The primers for amplification were listed as follows; the forward primer 5′-TCTTCCATTTCAGGTGTCGTGAACACGC-3′, and the reverse primer 5′-GCGGCCGCCCAGCTCGTCCATGCCGAGAGT-3′.

### Mathematical modeling of gene recombination and statistical analysis

A mathematical model of recombination between the *YFP* and *CFP* genes was built as follows: First, the *CFP* (*nTurquoise-GL)* gene was recombined randomly with the identical nucleotide of *hYFP*, but not *eYFP*, according to the recombination rate, *r* (/base). If the reverse-transcript of *CFP* is recombined with *hYFP* between the first nucleotide and nt 199, the recombinant gene product emits CFP fluorescence, because the Y66W mutation is responsible for the cyan fluorescence. If *CFP* is recombined with *hYFP* after nt 198, the chimeric GFP or YFP gene product emits yellow fluorescence. Depending on the recombination site, fluorescence intensity of chimeric GFP gene reduces based on the experimental results in Supplementary Figure S2B. To recapitulate the experimental data set, fluorescence intensities and deviations for intercellular heterogeneity were obtained from the experimental data, and applied to simulation (Supplementary Fig. S6). The parameter *r* was obtained by the maximal likelihood estimation method. Given *r*, the log likelihood value was calculated as follows


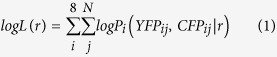


where *P*_*i*_ is a probability density obtained by simulation with the given *r* value, and *YFP*_*ij*_ and *CFP*_*ij*_ are YFP and CFP fluorescence intensities at the *j*-th cell in experimental data of the *i*-th condition among eight different *YFP* genes. To obtain *P*, the recombination events were repeated in at least 30,000 cells under each condition. In computer simulation, the value of *r* was varied in the range of 0.0001–0.01 to obtain the *r* value showing maximal log likelihood value.

## Additional Information

**How to cite this article**: Komatsubara, A. T. *et al.* Quantitative analysis of recombination between YFP and CFP genes of FRET biosensors introduced by lentiviral or retroviral gene transfer. *Sci. Rep.* **5**, 13283; doi: 10.1038/srep13283 (2015).

## Supplementary Material

Supplementary Information

## Figures and Tables

**Table 1:** Recombination rates calculated by the mathematical model.

	Recombination rate (/base)	
	Lentivirus	Retrovirus
A549 cells	0.0031	0.0024
HeLa cells	0.0044, 0.0021*	0.0021

^*^Recombination rate of a FRET biosensor with YPet, GGSGG linker, nTurquoise-GL, and NES in Fig. 5B.
