# Supplementary material for: Quantitative analysis of recombination between YFP and CFP genes of FRET biosensors introduced by lentiviral or retroviral gene transfer
Source: Sci Rep. 2015 Aug 20;5:13283. doi: 10.1038/srep13283 (PMC4542544; doi:10.1038/srep13283)
Supplement: Supplementary Information [file srep13283-s1.pdf]

Supplementary Information:

Quantitative analysis of recombination in YFP and CFP gene of FRET biosensor induced by lentiviral or retroviral gene transfer.

Akira T. Komatsubara,[1] Michiyuki Matsuda,[1,2] and Kazuhiro Aoki[3,*]

[1] Laboratory of Bioimaging and Cell Signaling, Graduate School of Biostudies, Kyoto University, Sakyo-ku, Kyoto 606-8501, Japan
[2] Department of Pathology and Biology of Diseases, Graduate School of Medicine, Kyoto University, Sakyo-ku, Kyoto 606-8501, Japan
[3] Imaging Platform for Spatio-Temporal Information, Graduate School of Medicine, Kyoto University, Sakyo-ku, Kyoto 606-8501, Japan

Supplementary Figure S1

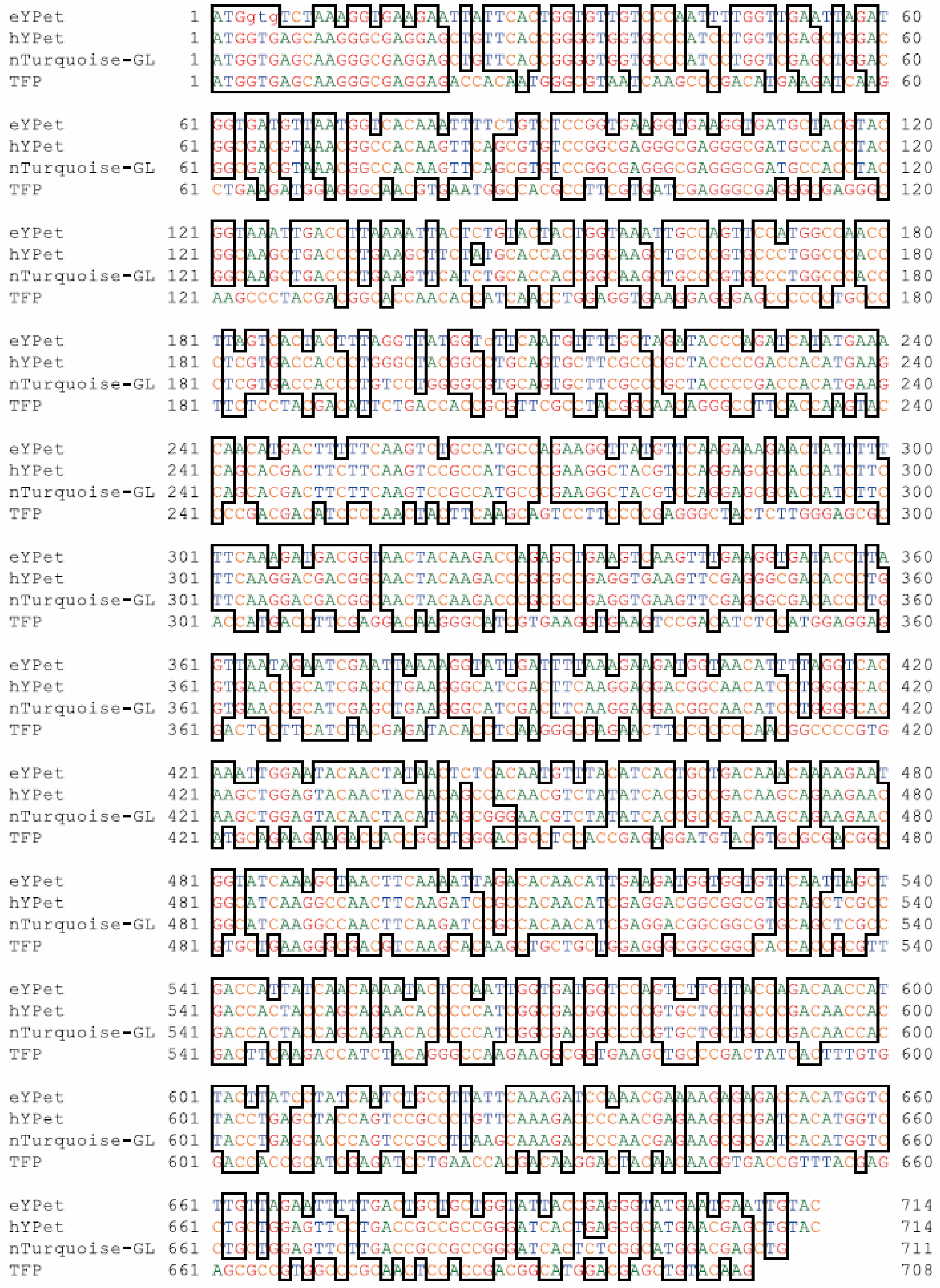

FIGURE S1 Multiple alignment of nucleotide sequences in *YFP* and *CFP* genes. DNA sequences of *e100YPet*, *h100YPet*, *nTurquoise-GL*, and *TFP* were aligned to highlight their sequence identity with the boxed area.

Supplementary Figure S2

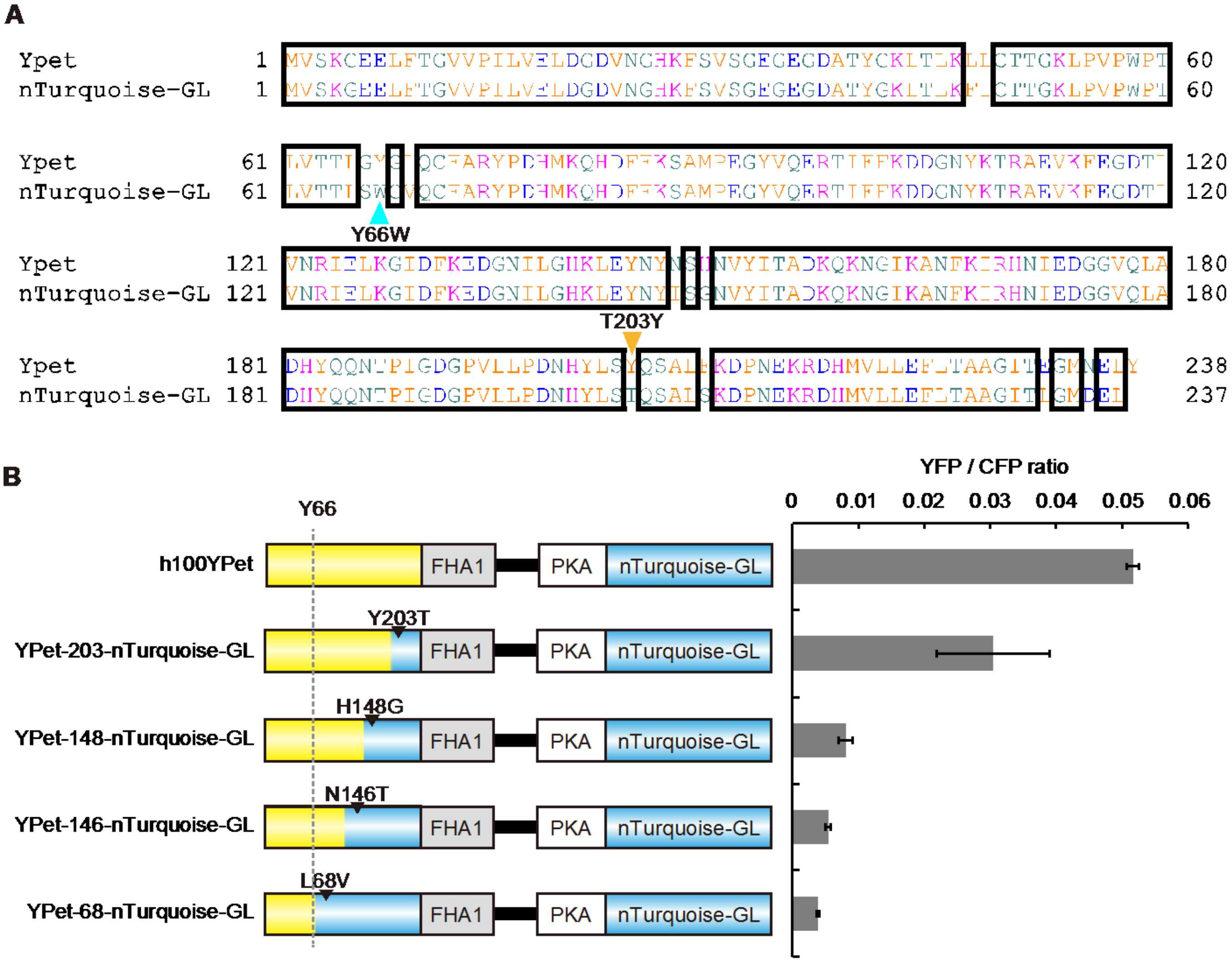

FIGURE S2 Comparison of fluorescence intensities among chimeric GFP variants. (A) An alignment of amino acid sequences in YPet and nTurquoise-GL. (B) Fluorescence intensities of h100YPet, chimeric GFP variants and CFP were measured in HeLa cells transiently expressing the indicated biosensors (Left). Fluorescence intensities of YFP channel were normalized by fluorescence intensities of CFP channel. The averaged YFP/CFP ratios are represented with S.E. (N > 50 cells).

Supplementary Figure S3

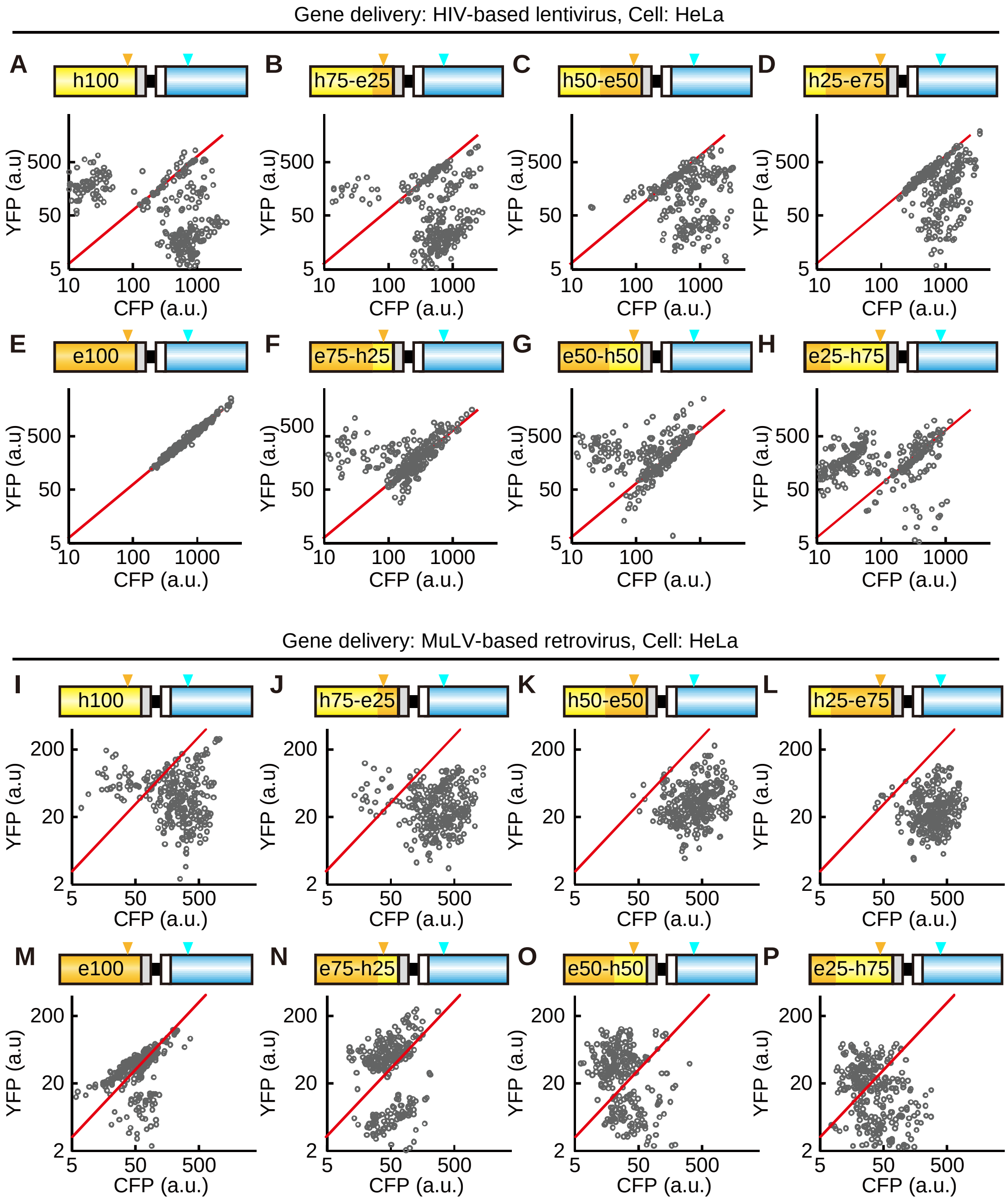

FIGURE S3 Recombination between *YFP* and *CFP* genes by lentiviral or retroviral gene transfer in HeLa cells. HeLa cells were infected with lentivirus (A-H) or retrovirus (I-P) encoding 8 different FRET biosensors as shown in the upper panel. At least 4 days after infection, the cells were imaged with an epi-fluorescence microscope. The average fluorescence intensities of CFP and YFP are represented as a log-log plot. Each dot corresponds to a HeLa cell. Three hundred cells were analyzed from two independent experiments. Red lines are the fitted line with the *e100YPet* data. Orange and cyan arrowheads indicate the T203Y and Y66W mutations, respectively.

Supplementary Figure S4

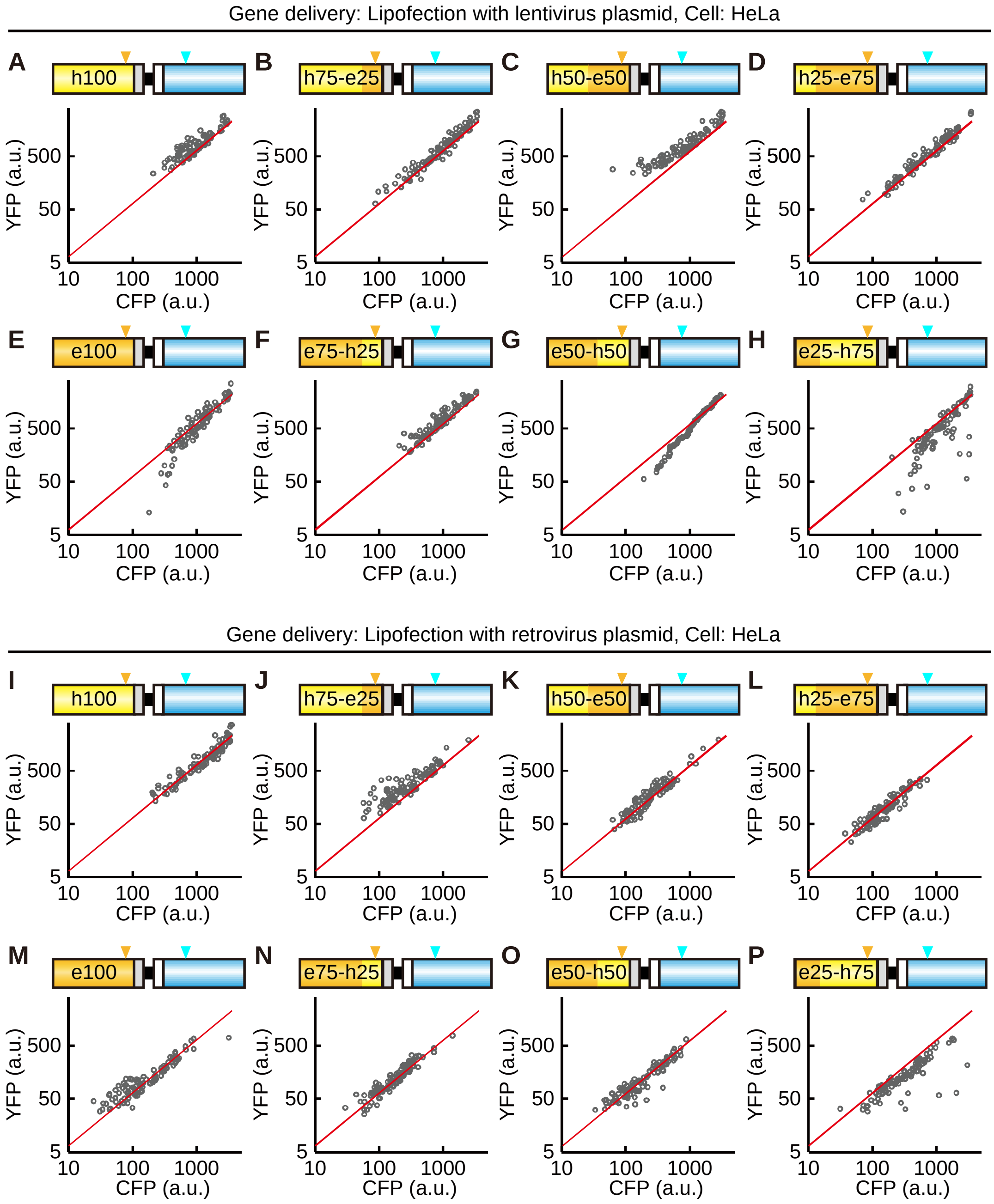

FIGURE S4 Transient expression of YFP and CFP in HeLa cells with lipofection. HeLa cells were transfected with lentiviral (A-H) or retroviral (I-P) vector plasmids encoding 8 different FRET biosensors as shown in the upper panel. At least 2 days after transfection, the cells were imaged with an epi-fluorescence microscope. The average fluorescence intensities of CFP and YFP are represented as a log-log plot. Each dot corresponds to a HeLa cell. Three hundred cells were analyzed from two independent experiments. Red lines are the fitted line with the *e100YPet* data. Orange and cyan arrowheads indicate the T203Y and Y66W mutation, respectively.

Supplementary Figure S5

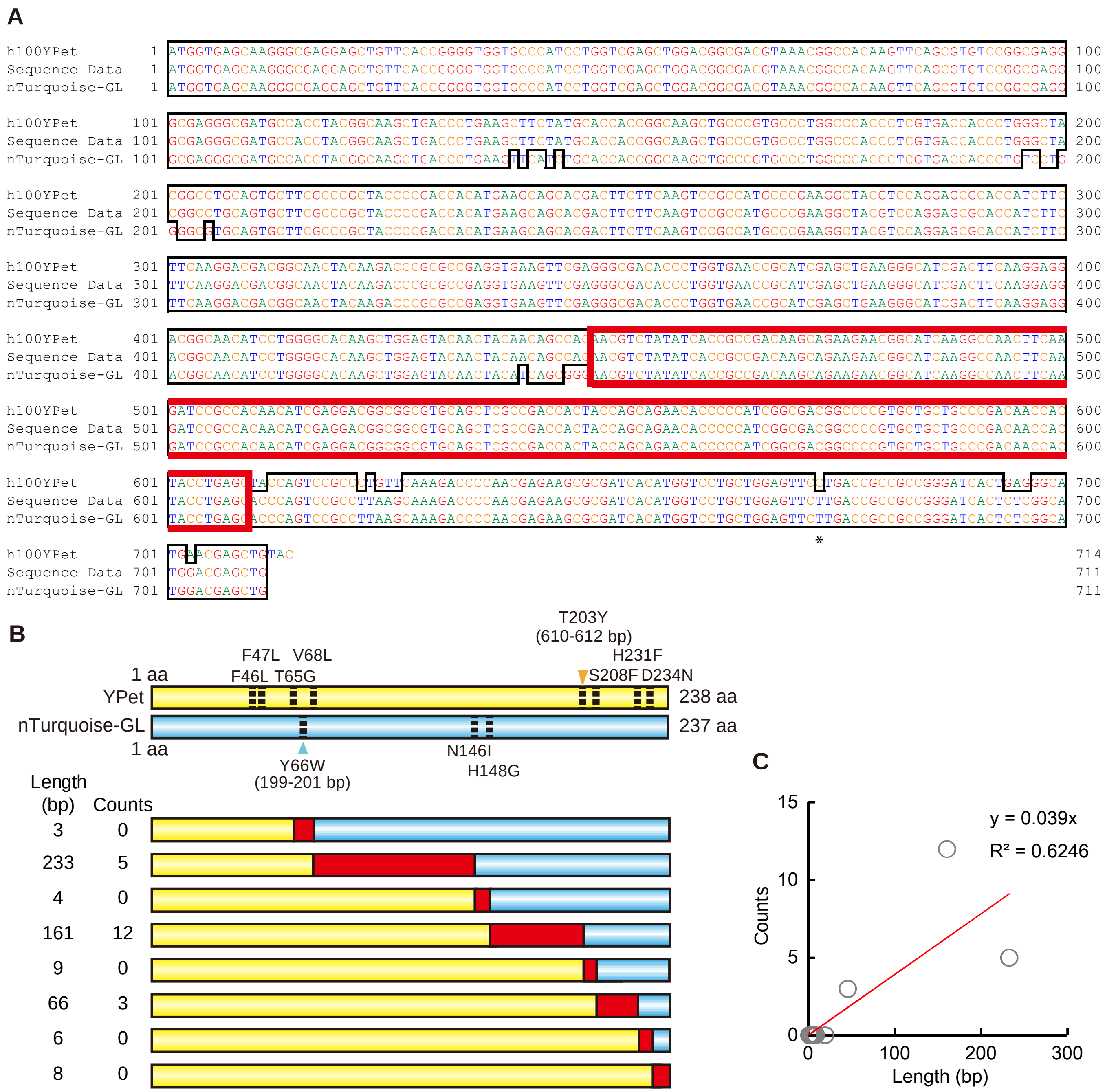

FIGURE S5 Verification of the recombination. (A) An example of results of DNA sequencing in recombined *YPet* and *nTurquoise-GL* genes. A549 cells infected with *h100YPet*-carrying lentivirus were sorted by FACS depending on YFP fluorescence, followed by genomic DNA extraction, PCR amplification of the recombined genes, and sequencing. Nucleotide sequences of wild-type *h100YPet*, a representative recombined DNA sequence, and wild-type *nTurquoise-GL* are aligned with black lines, which enclose identical nucleotides. The red line encloses the region where the recombination has

occurred. *, synonymous mutation. (B) Schematic representation of the difference between YPet and nTurquoise-GL amino acids. (Upper) The dotted lines represent different amino acid residues between YPet and nTurquoise-GL. (Lower) We analyzed chimeric GFP and YFP variants, in which Y66 amino acid was conserved. Red regions indicate potential recombination site. Length indicates the number of identical DNA sequence for each potential recombination sites. Counts indicate the number of recombined *GFP* or *YFP* variants that have been generated as a result of recombination in between the identical DNA sequence. (C) The number of recombined *GFP* or *YFP* variants are plotted as a function of the number of each identical DNA sequence, in which the recombination has occurred.

Supplementary Figure S6

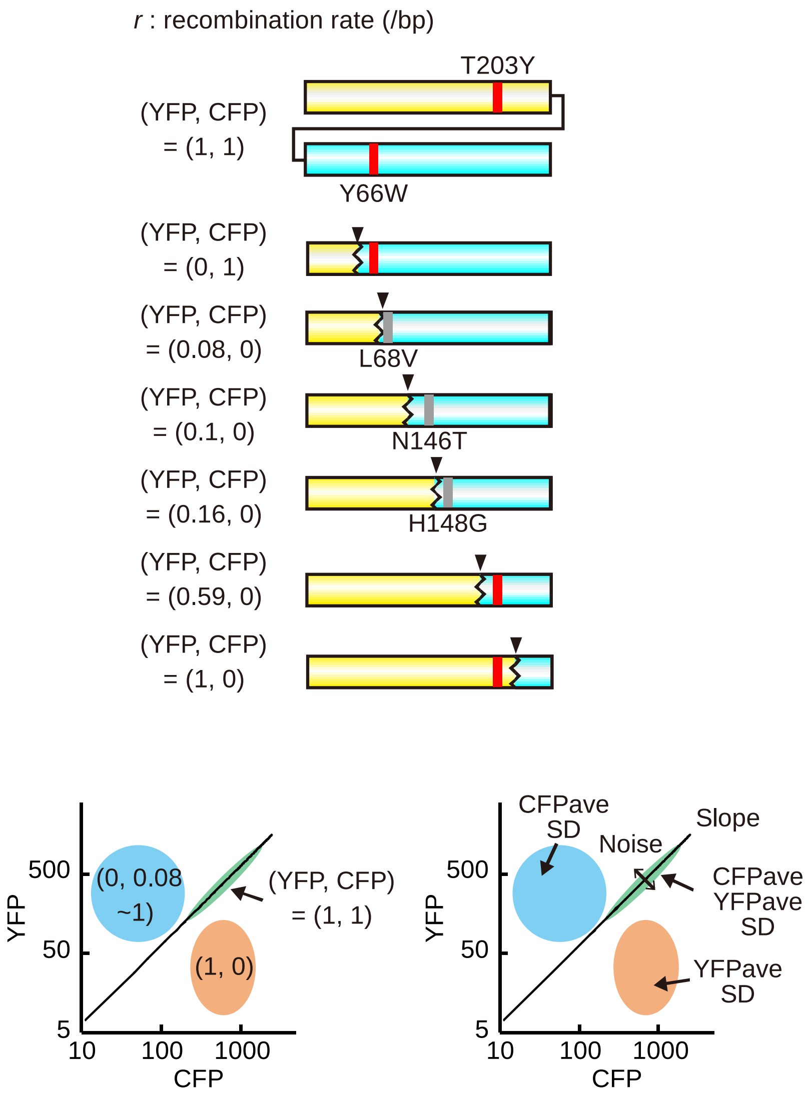

FIGURE S6 Scheme of the mathematical model of recombination between *YFP* and *CFP* genes. (Upper panel) Schematic representation of the recombination of *YFP* and *CFP* genes. Red lines indicate the critical amino acid residues for the substitution of CFP and YFP from GFP, Y66W and T203Y, respectively. Black arrowheads indicate the position of recombination. Of note, fluorescence intensity of chimeric GFP genes is changed in accord with the recombination sites (Supplementary Fig. S2B). (Lower panels) The distribution of YFP and CFP intensities are illustrated in a log-log plot. The blue, orange, and green areas represent cells expressing CFP, YFP, or both, respectively (left). To recapitulate the experimental data, the indicated 9 parameters were extracted from the experimental data set (right).

Supplementary Figure S7

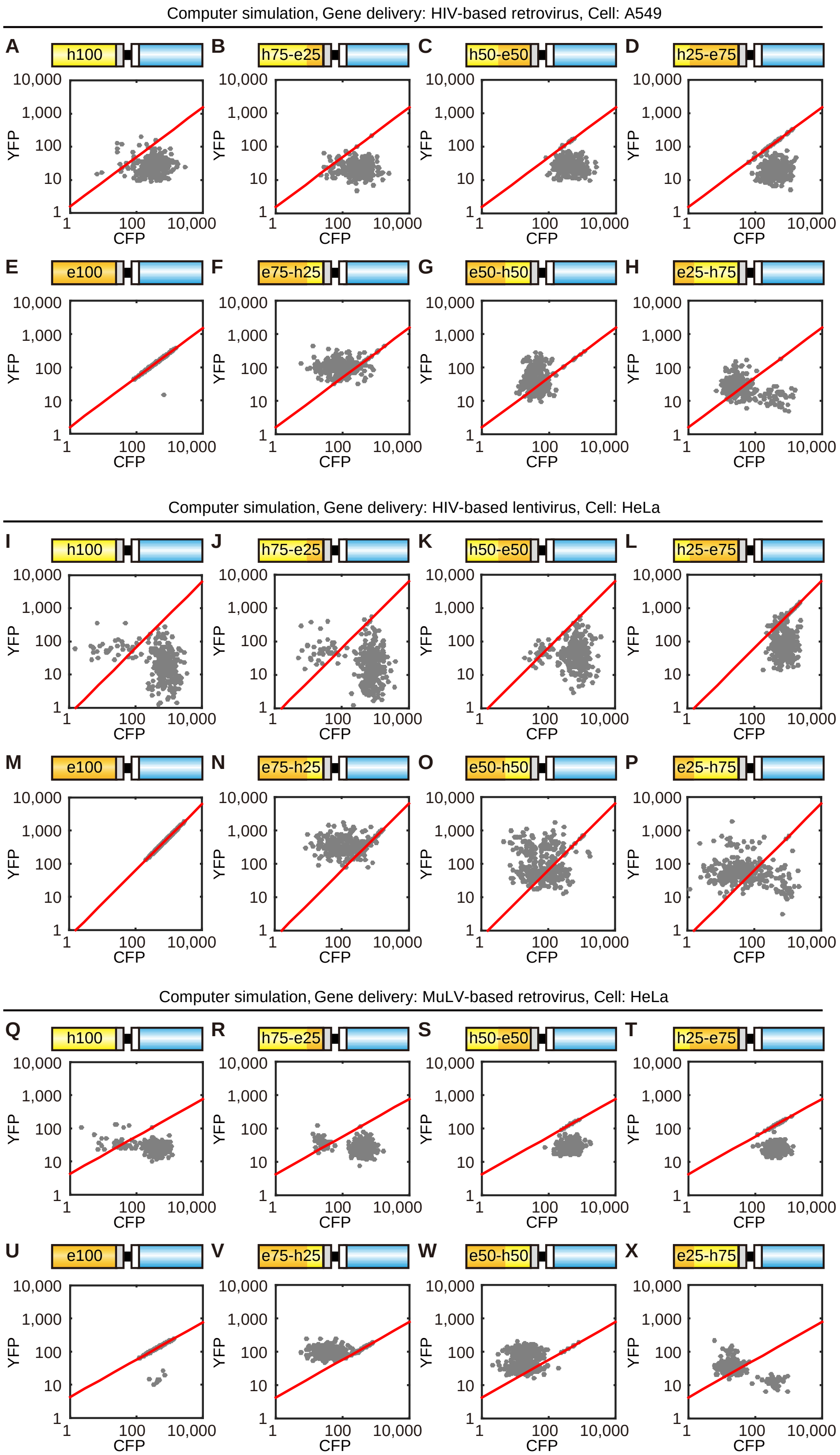

FIGURE S7 Computer simulation of the recombination between *YFP* and *CFP* genes. The recombination of *YFP* and *CFP* genes in A549 cells infected with the indicated retrovirus (A-H) or HeLa cells infected with the lentivirus (I-P) or retrovirus (Q-X) was simulated by computer with the recombination rates, which showed maximal likelihood estimation (Table 1). To reproduce the experimental data, 9 parameters were extracted from the experimental data set in Figure 3, and Supplementary Figure S3 (see Supplementary Figure S6 and the Methods for details). Red lines are the fitted line with the *e100YPet* data. Orange and cyan arrowheads indicate the T203Y and Y66W mutations, respectively.

Supplementary Figure S8

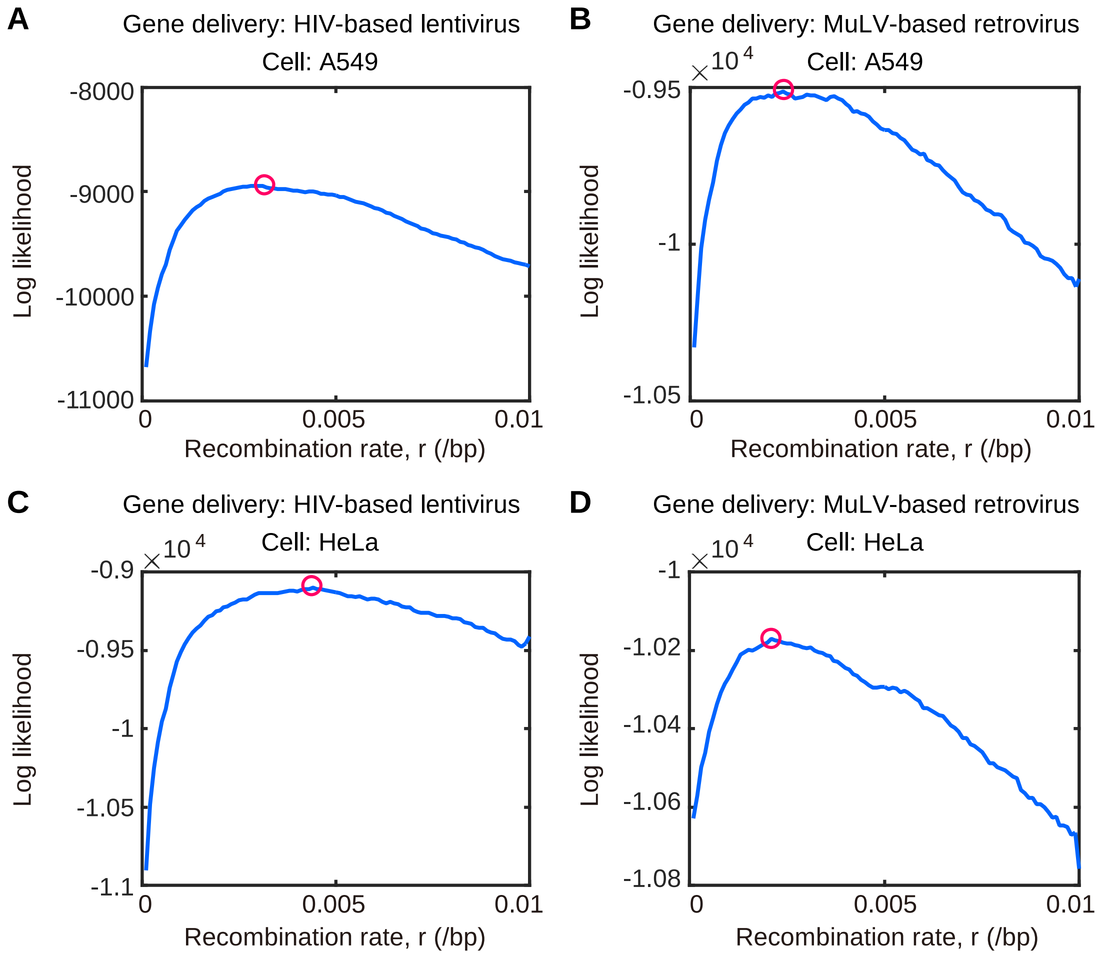

FIGURE S8 Maximal log-likelihood estimation of recombination rate. Log-likelihood values were calculated as described in Methods, and plotted as a function of recombination rate (r) in A549 cells infected with lentivirus (A) or retrovirus (B), or in HeLa cells infected with lentivirus (C) or retrovirus (D). The red circles indicate maximal log-likelihood values.
